# SMT19969 for *Clostridium difficile* infection (CDI): *in vivo* efficacy compared with fidaxomicin and vancomycin in the hamster model of CDI

**DOI:** 10.1093/jac/dkv005

**Published:** 2015-02-03

**Authors:** Abdul Sattar, Pia Thommes, Lloyd Payne, Peter Warn, Richard J. Vickers

**Affiliations:** 1 Evotec (UK), Williams House, Manchester Science Park, Lloyd Street North, Manchester M15 6SE, UK; 2 Summit plc, Abingdon, Oxfordshire, UK

**Keywords:** *C. difficile*, treatment, antimicrobial

## Abstract

**Objectives:**

SMT19969 is a novel narrow-spectrum antimicrobial under development for the treatment of *Clostridium difficile* infection (CDI). The objectives were to assess the relative efficacies of SMT19969, vancomycin and fidaxomicin in the hamster model of CDI.

**Methods:**

Hamsters were infected with either *C. difficile* BI1 (ribotype 027) or *C. difficile* 630 (ribotype 012) prior to treatment with vehicle, SMT19969, fidaxomicin or vancomycin for 5 days. Animals were further monitored through to day 28 and survival recorded. Plasma and gastrointestinal concentrations of SMT19969 following single and repeat administration in infected hamsters were determined.

**Results:**

Following infection with *C. difficile* BI1, treatment with SMT19969, vancomycin and fidaxomicin resulted in 100% survival during the 5 day dosing period, with 90%–100% of animals receiving SMT19969 and fidaxomicin surviving during the post-dosing follow-up period. Whilst protective during treatment, onset of mortality was observed on day 11 in animals treated with vancomycin, with a 10% survival recorded by day 28. Similar results were observed for SMT19969 and vancomycin following infection with *C. difficile* 630, with day 28 survival rates of 80%–100% and 0%, respectively. Fidaxomicin protected animals infected with *C. difficile* 630 from mortality during dosing, although day 28 survival rates varied from 0% to 40% depending on dose. Plasma levels of SMT19969 were typically below the limit of quantification, but levels in the gastrointestinal tract remained far in excess of the MIC.

**Conclusions:**

These data show that SMT19969 is highly effective at treating both acute infection and preventing recurrent disease and support continued investigation of SMT19969 as a potential therapy for CDI.

## Introduction

Over the past two decades, the emergence of hypervirulent epidemic strains of *Clostridium difficile* has been associated with increasing prevalence and severity of disease with a recognition of disease occurring in the community and primary care facilities.^1,2^

Although the molecular epidemiology of *C. difficile* infection (CDI) differs across geographical locations, the hypervirulent ribotype 027 (BI/NAP1) remains the predominant strain in the USA,^3^ accounting for approximately one-third of cases and continues to be the most commonly isolated ribotype in many Central and Eastern European countries.^4^

Conventional agents (metronidazole and vancomycin) are associated with high rates of recurrent disease.^5^ Fidaxomicin, which received approval in 2011, has been shown to be non-inferior to vancomycin on clinical response at end of therapy but superior to vancomycin in sustaining clinical response to 25 days post end of therapy.^6,7^ Currently, there is little supporting clinical evidence for the use of fidaxomicin in subjects suffering multiple recurrences of CDI.^8^ In addition, metronidazole is inferior to vancomycin in treating severe CDI.^9^

SMT19969 is a novel non-absorbable antibiotic in Phase 2 clinical development for the treatment of CDI. With a narrow spectrum of activity and high selectivity for *C. difficile* over Gram-positive and Gram-negative anaerobic and facultative faecal flora,^10,11^ SMT19969 has therapeutic potential for the treatment of CDI.

Here, we report the results from studies in an established hamster model^12^ comparing the efficacy of SMT19969, fidaxomicin and vancomycin following infection with either *C. difficile* ribotype 027 or 012. In addition, plasma and gastrointestinal (GI) concentrations of SMT19969 following single and repeat administration in infected hamsters were determined.

## Methods

### Regulatory

Animal experiments were performed under UK Home Office ASPA Licence 40/3644 and with ethics committee clearance (the University of Manchester Committee). All experiments were performed by technicians who have completed parts 1–3 of the Home Office Personal Licence course and hold current personal licences.

### Animal strain

Golden Syrian hamsters used in these studies were supplied by Janvier Laboratories (France) and were specific pathogen free. Hamsters were housed in sterile individual ventilated cages exposing hamsters at all times to HEPA-filtered sterile air. Hamsters had free access to food and water and sterile aspen chip bedding.

### Bacterial isolates

*C. difficile* strains BI1 (ribotype 027) and 630 (ribotype 012) used in these studies were supplied by B. Wren, London School of Hygiene and Tropical Medicine, UK.

### Pre-infection

Hamsters were identified by subcutaneous implantation of temperature-recording identification chips (Plexx IPPT 300 programmable non-contact temperature transponders). Hamsters were rendered susceptible to infection by *C. difficile* by administration of 30 mg/kg oral clindamycin (Villerton, Luxembourg) 24 h prior to infection.

### Infection

All hamsters were infected with ∼100–350 *C. difficile* spores by oral gavage 24 h post-clindamycin administration.

### Preparation of test articles and dosing

SMT19969 (manufactured to Good Manufacturing Practice guidelines by CML, Weert, The Netherlands) was first suspended in 50 mg/mL DMSO (Sigma) and then further diluted in aqueous 0.5% methyl cellulose to provide a suspension for administration. Fidaxomicin (Dificlir, 200 mg film-coated tablets, Astellas Pharma Europe) was ground to a powder, suspended in 20 mg/mL DMSO and then further diluted in aqueous 0.5% methyl cellulose to provide a suspension for administration. Vancomycin (Vancocin^®^, Flynn Pharma, Stevenage, UK) was freshly prepared once daily from a 50 mg/mL stock solution made up in water for injection. The 50 mg/mL stock solution was diluted in physiological saline for dosing solutions. Vehicle-treated animals were administered aqueous 0.5% methyl cellulose. All solutions were prepared once daily and stored at 4°C between doses. Animals were treated at 10 mL/kg by oral gavage. Treatment was initiated 20 h post-infection and administered twice daily for 5 days using a sterile plastic disposable 1.5 mm diameter (4.5 FG) dosing catheter.

### In-life sample collection

Faecal samples were collected from all surviving animals on days 1, 7, 12, 19 and 28 post-infection. Faecal samples were cultured for the presence of *C. difficile* spores after treatment with 70% ethanol for 5 min.

### Endpoints

The efficacy of the test agents was assessed by monitoring and comparing daily survival rates. The hamsters were monitored at a frequency appropriate to their clinical condition. Hamsters that developed hypothermia (<33°C), diarrhoea, weight loss (>20%) or other signs of severe disease were euthanized by an overdose of pentobarbitone. As soon as death was confirmed, the abdomen of the hamsters was dissected and the presence or absence of megacolon and any other obvious features or pathology was recorded. Samples of the distal small intestine (ileum) contents, caecum contents and colon contents were collected for culture. Anaerobic cultures of the gut contents were established on selective media to assess the *C. difficile* vegetative bacteria and spore burdens (material cultured for spore burden was exposed for 5 min to 70% ethanol). All surviving animals were euthanized 28 days post-infection (5 days of dosing and 23 days of observation) and *C. difficile* vegetative bacteria and spore burdens were determined in small intestine (ileum) contents, caecum contents and colon contents.

### Pharmacokinetics

At the appropriate timepoints, hamsters were administered an overdose of pentobarbitone. When animals were deeply unconscious, blood was collected into a heparinized syringe by cardiac puncture. The blood samples were placed on ice immediately after collection and centrifuged as soon as possible at 3000 rpm for 5 min. The plasma was removed and stored at −80°C until shipment to CEM Analytical Services (CEMAS, Wokingham, Berkshire, UK). As soon as death was confirmed, the abdomen of the animals was opened and the entire gut carefully opened lengthwise. A portion of the contents of the stomach, upper small intestine, caecum and colon was removed and immediately frozen at −80°C until shipment to CEMAS for SMT19969 quantification. Bioanalysis of the plasma and gut content samples for SMT19969 was performed by CEMAS using LC-MS/MS. The lower limit of quantification (LOQ) was 1 ng/mL for both plasma and GI samples.

## Results

The comparative efficacies of orally administered SMT19969, vancomycin, fidaxomicin and vehicle were assessed in hamsters following infection with either *C. difficile* strain BI1 (ribotype 027) or 630 (ribotype 012). All animals that were euthanized during the course of the study were considered to have succumbed to CDI following macroscopic examination of the GI tract on necropsy and positive culture of *C. difficile* from the GI contents. Animals treated with vehicle and study drug that succumbed due to CDI all showed significant inflammation of the GI tract, which was particularly apparent in the ileum. Animals that survived to the end of the study showed no inflammation of the GI tract on necropsy.

SMT19969 was assessed at two different doses (12.5 and 25 mg/kg) with twice-daily dosing. SMT19969 dose level and regimen selection was based on published data^13^ and preliminary dose-ranging studies carried out as part of the studies described here (data not shown). Based on published data on the efficacy and dosing of fidaxomicin in the hamster model of CDI,^14,15^ treatment regimens of 1 and 2.5 mg/kg twice daily were assessed following infection by *C. difficile* strains BI1 and 630 with additional regimens of 12.5 and 25 mg/kg twice daily used following infection with *C. difficile* 630.

### Hamster survival, ribotype 027

**Figure 1.**
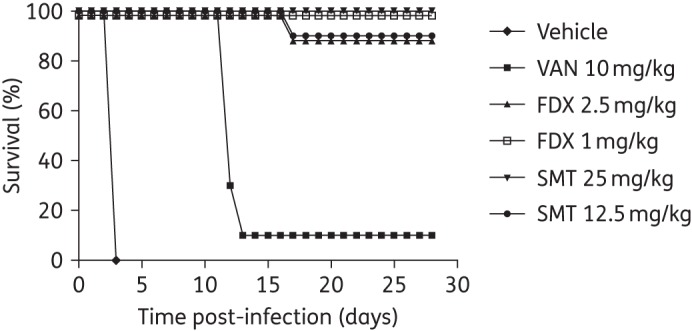
Daily survival (%) following infection with *C. difficile* BI1. VAN, vancomycin; FDX, fidaxomicin; SMT, SMT19969.

A severe model of CDI was established following infection with *C. difficile* BI1 with survival data shown in Figure 1. All vehicle-treated animals succumbed to infection (100% mortality) with the deaths occurring by 72 h post-infection. On day 11 post-infection (6 days following cessation of treatment), 7 of 10 hamsters administered 10 mg/kg vancomycin twice daily developed CDI and were euthanized with a further 2 vancomycin-treated hamsters euthanized due to severe diarrhoea and hypothermia on day 12 post-infection. A single animal treated with 10 mg/kg vancomycin twice daily survived until the end of the study.

On day 16 post-infection (11 days following cessation of treatment), one hamster treated with 2.5 mg/kg fidaxomicin twice daily and another treated with 12.5 mg/kg SMT19969 twice daily were euthanized due to significant cumulative weight loss and the development of CDI. All other animals in these treatment groups and all animals treated with either 1 mg/kg fidaxomicin twice daily or 25 mg/kg SMT19969 twice daily survived until the end of the study.

### Hamster survival, ribotype 012

**Figure 2.**
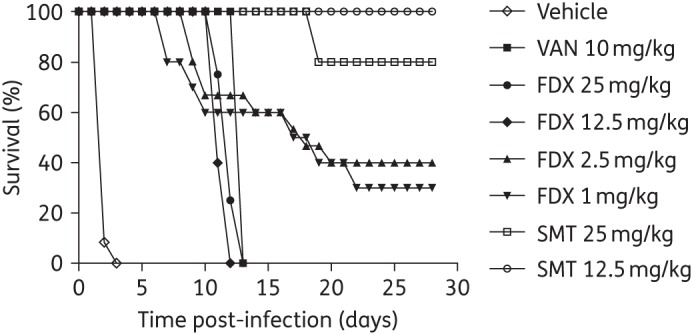
Daily survival (%) following infection with *C. difficile* 630. VAN, vancomycin; FDX, fidaxomicin; SMT, SMT19969.

The comparative efficacy of the test agents was also assessed in hamsters infected with *C. difficile* 630 (Figure 2). As discussed below, an unexpectedly high level of mortality due to CDI was observed in animals treated with 1 or 2.5 mg/kg fidaxomicin during the post-treatment observation period. To address the potential for a suboptimal dosing regimen of fidaxomicin for this strain, an additional study was conducted that included higher doses (12.5 and 25 mg/kg twice daily) of fidaxomicin and the results discussed are a pooled analysis of these studies based on the statistically indistinguishable outcomes of treatment in groups that were included in both models.

As observed for infection with *C. difficile* BI1, infection with *C. difficile* 630 resulted in a severe model of CDI with 100% mortality observed in vehicle-treated animals by day 3 post-infection. Vancomycin-treated animals all survived the course of dosing with rapid onset of disease observed during the post-treatment follow-up period with 100% mortality observed on day 13.

SMT19969 conferred significant protection from CDI during both the initial dosing and follow-up periods. At a dose of 12.5 mg/kg twice daily, 100% survival was observed during dosing and through to the end of study (day 28). Administration of SMT19969 at a dose of 25 mg/kg twice daily resulted in 100% survival during dosing with mortality onset at day 18 resulting in 80% survival, which was maintained to the end of the study.

All doses of fidaxomicin conferred protection from CDI during the course of dosing, although onset of mortality was observed between days 6 and 8 for animals administered 1 or 2.5 mg/kg twice daily with a gradual loss of animals observed through to day 22 resulting in 30% and 40% survival, respectively, which was maintained to day 28. Higher doses of fidaxomicin (12.5 and 25 mg/kg twice daily), whilst protecting from CDI during the course of dosing, resulted in high mortality during the follow-up period with mortality starting on day 10 resulting in 0% survival on days 12 and 13, respectively.

### Spore counts

**Figure 3.**
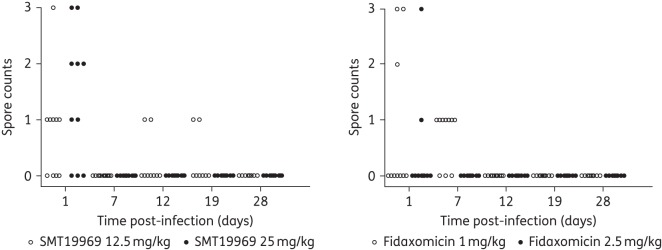
Semi-quantitative spore counts for faecal samples recovered from individual animals post-infection with *C. difficile* BI1. Spore counts: 1, ≤10 colonies; 2, 11–100 colonies; and 3, >100 colonies.

**Figure 4.**
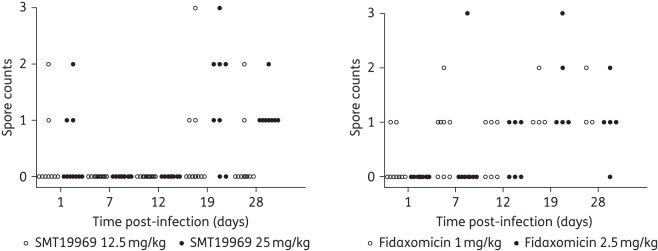
Semi-quantitative spore counts for faecal samples recovered from individual animals post-infection with *C. difficile* 630. Spore counts: 1, ≤10 colonies; 2, 11–100 colonies; and 3, >100 colonies.

Faecal samples were collected from treated animals on days 1, 7, 12, 19 and 28 post-infection for semi-quantitative culture of *C. difficile* spores (Figures 3 and 4).

Following infection with *C. difficile* ribotype 027 (BI1), spores were isolated on day 1 for 13 of 20 animals administered SMT19969 and 5 of 20 animals administered fidaxomicin. For animals administered the lower doses of either SMT19969 (12.5 mg/kg) or fidaxomicin (1 mg/kg), spores were isolated from two animals administered SMT19969 from samples collected on days 12 and 19 and in seven animals administered fidaxomicin from samples collected on day 7. At the higher doses of SMT19969 (25 mg/kg) and fidaxomicin (2.5 mg/kg), no spores were recovered from any faecal sample from day 7 onwards.

For animals infected with *C. difficile* ribotype 012 (630), faecal samples from animals administered SMT19969 were negative for spores on days 7 and 12 post-dosing, although a significant proportion of samples were spore positive on days 19 and 28 post-infection. For animals administered fidaxomicin, the majority of faecal samples on days 12, 19 and 28 post-infection were positive for spores. Day 1 and 7 faecal samples (with the exception of a single animal) following administration of 2.5 mg/kg fidaxomicin were spore negative, although at the lower dose of fidaxomicin (1 mg/kg), 2 of 10 and 5 of 8 surviving animals were positive for spores on days 1 and 7 post-infection, respectively.

Faecal samples from animals treated with either 12.5 or 25 mg/kg fidaxomicin twice daily were not assessed for the presence of spores, although the colon and caecum contents of all animals that succumbed to CDI were positive for the presence of *C. difficile* vegetative cells and spores at the time of euthanasia (data not shown).

### Pharmacokinetics

Following oral administration of either 12.5 or 25 mg/kg SMT19969, plasma concentrations of SMT19969 at all timepoints were typically below the LOQ (1 ng/mL), indicating very low absorption from the GI tract of infected hamsters. SMT19969 was quantified in isolated plasma samples (two animals at 8 h post 12.5 mg/kg dose, one animal at 1 h post 25 mg/kg dose and one animal at 8 h post 25 mg/kg dose), although concentrations were very low, ranging from 1.3 to 7.1 ng/mL.

**Figure 5.**
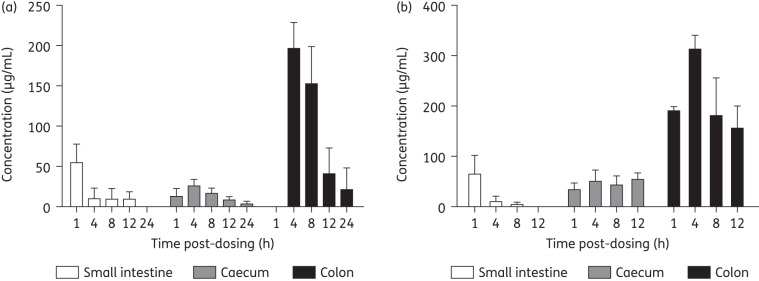
Mean concentrations (+SD) of SMT19969 in sections of the GI tract following (a) single or (b) two doses at 25 mg/kg.

Data for the concentrations of SMT19969 in sections of the GI tract following either a single or two (12 h apart) oral doses of 25 mg/kg SMT19969 are shown in Figure 5 (data for stomach contents not shown in Figure 5 for clarity). Following a single dose, mean levels of SMT19969 in the stomach and small intestine peaked at 2.8 and 55.1 μg/mL, respectively, and were largely cleared by 1 or 4 h post-dose, respectively. Mean levels of SMT19969 in caecum contents peaked at 25.2 μg/mL 4 h post-dose and persisted slightly longer than in the stomach and small intestine, falling to 8.3 μg/mL 12 h post-dose. Mean levels of SMT19969 were significantly higher in colon contents than other sections of the GI tract, peaking at 195.7 μg/mL 4 h post-dose, with significant levels of drug (mean = 20.9 μg/mL) persisting to 24 h post-dose.

Following a second dose of SMT19969 12 h later, a similar profile in stomach and small intestine contents was observed with mean levels of drug falling to <10 μg/mL by 4 h post-dose. However, consistent with significant levels of SMT19969 persisting to 24 h following a single dose, enhanced exposure was observed in caecum and colon contents following a second dose. Mean peak SMT19969 levels were ∼2-fold higher in caecum than levels recorded following a single dose and significant levels of SMT19969 (53.0 μg/mL) persisted to 12 h post-dose. Mean peak SMT19969 levels of 312.4 μg/mL were observed in the colon 4 h post-dose and were ∼1.5-fold higher than following a single dose. In addition, significant drug levels (189.1 μg/mL) were observed in the colon 1 h post-dosing and remained higher than following a single dose to 12 h post-dosing.

## Discussion

The hamster model of clindamycin-induced CDI is the current standard *in vivo* model used to assess the potential efficacy of agents including antibiotics, toxin antibodies and vaccines.^16^ This model has also been used to assess virulence and disease pathogenesis of CDI.^17,18^ Replicating many of the features of CDI in humans, the model has been used to confirm that toxins A and B and binary toxin (*C. difficile* toxins) all play important roles in virulence and disease severity.^19^

SMT19969 is a novel antibiotic, in clinical development for the treatment of CDI, with potent growth inhibition of *C. difficile* and a narrow spectrum of activity that may minimize further collateral damage to the gut microbiota during CDI therapy. Susceptibility testing in previous studies has shown excellent inhibitory activity against *C. difficile* with an MIC_90_ value of 0.25 mg/L, which was 2–4-fold more potent than those of either metronidazole or vancomycin and 2-fold more active than fidaxomicin.^11^

Following infection with *C. difficile* BI1, both SMT19969 and fidaxomicin conferred significant protection from CDI with 100% survival recorded during the course of dosing and a 90%–100% survival rate observed during the post-treatment follow-up period (Figure 1). As expected, vancomycin-treated animals survived the course of dosing with onset of mortality observed on day 11 with only a 10% survival rate recorded by day 28.

SMT19969 administered at 12.5 and 25 mg/kg twice daily was associated with improved survival rates when compared with vehicle-, vancomycin- or fidaxomicin-treated hamsters following infection with *C. difficile* 630 (Figure 2). Although all doses of fidaxomicin resulted in 100% survival during the course of dosing, recurrent disease following withdrawal of treatment was observed with a 0%–40% survival recorded by day 28. Higher doses of fidaxomicin (12.5 and 25 mg/kg twice daily) did not result in increased protection from disease. This observation was unexpected based on the previously reported efficacy of fidaxomicin in the hamster model of CDI,^14,15^ although the high variability of dose regimens and experimental methods used in the hamster model can make comparison between studies problematic.^16^ However, a significant number of faecal samples collected during the course of the study (days 1, 7, 12, 19 and 28 post-infection) were positive for *C. difficile* spores (Figure 4) following administration of fidaxomicin whereas samples were spore negative on days 7 and 12 following administration of SMT19969. Further studies are warranted to investigate and confirm the unexpectedly high levels of recurrent disease seen with fidaxomicin against *C. difficile* 630.

Data presented here confirm that SMT19969 is minimally systemically absorbed, even in the presence of GI inflammation due to CDI, with plasma concentrations typically below the LOQ (1 ng/mL). Following a single dose of 25 mg/kg, mean concentrations of SMT19969 in small intestine, caecum and colon samples of 9.70, 8.26 and 40.46 μg/mL, respectively, were recorded at 12 h post-dosing, which were significantly in excess of the typical *C. difficile* MIC value of 0.125–0.25 mg/L. A second dose administered after 12 h resulted in increased drug concentrations in the caecum and colon (53.0 and 155.3 μg/mL at 12 h post-dosing, respectively) and these data indicate that twice-daily dosing increased both the peak drug concentration and the time the drug concentration was significantly (∼100-fold) in excess of MIC. This was most apparent in the colon, the site of infection in humans. These observations following twice-daily dosing may contribute to the reduced rates of mortality observed in these studies during the post-dosing observation period (following infection with ribotype 027) compared with both the previously reported studies^13^ and the preliminary dose-ranging studies carried out as part of this study (data not shown) where once-daily dosing was used.

All currently available therapies are generally effective at treating the initial period of CDI, although they are associated with high rates of recurrent disease.^5^ Phase 3 studies have demonstrated that fidaxomicin is associated with reduced rates of recurrence compared with vancomycin, although rates of recurrence were comparable for subjects infected with hypervirulent ribotype 027 strains.^6,7^ Fidaxomicin has been shown to inhibit *C. difficile* sporulation and is superior to vancomycin in inhibiting the outgrowth of vegetative cells from germinated spores.^20^ Spores are the most common vector by which *C. difficile* is transmitted and are able to persist in the environment for extended periods;^21,22^ inhibition of sporulation may therefore impact on rates of recurrent disease. In this study, animals administered SMT19969 remained culture negative for spores for longer and had a lower rate of relapse than those administered fidaxomicin, although further studies are needed to examine the effect of SMT19969 on sporulation. In addition, narrow-spectrum antibiotics, such as SMT19969 and fidaxomicin, may also be associated with an improvement in microbiota recovery time and subsequent return of colonization resistance.

The results of these studies, in conjunction with previously reported data,^13^ show that SMT19969 is highly effective against different strains of *C. difficile* in the hamster model of CDI with significant protection from mortality observed during both the acute infection and post-dosing observation periods. Although SMT19969 was detected at low levels in isolated plasma samples, the data confirmed that SMT19969 is largely restricted to the GI tract in infected animals and twice-daily dosing resulted in more favourable intraluminal GI drug concentrations. Furthermore, SMT19969 has demonstrated potent and selective inhibition of *C. difficile* and was shown to be safe and well tolerated in a Phase 1 clinical trial. Continued investigation of SMT19969 as a therapy for CDI is warranted.

## Funding

This study was initiated and financially supported by Summit plc through a Seeding Drug Discovery Award (grant number 091055) and a Translation Award from the Wellcome Trust (grant number 099444).

Editorial support provided by Innovative Strategic Communications, LLC, was funded by Summit plc.

## Transparency declarations

This study was performed as contract research by Evotec. A. S., P. T., L. P. and P. W. are employees of Evotec and R. J. V. is an employee of Summit plc and holds share options.

The editorial support of Innovative Strategic Communications, LLC, in the preparation of this manuscript is acknowledged.
